# The role of fibular for supramalleolar osteotomy in treatment of varus ankle arthritis: a biomechanical and clinical study

**DOI:** 10.1186/s13018-016-0462-2

**Published:** 2016-10-24

**Authors:** Hongmou Zhao, Xiaojun Liang, Yi Li, Guangrong Yu, Wenxin Niu, Yan Zhang

**Affiliations:** 1 Foot and Ankle Surgery Department, Honghui Hospital, Xi’an Jiaotong University College of Medicine, Xi’an, 710054 China; 2 Foot and Ankle Surgery Department, Tongji Hospital, Tongji University School of Medicine, Shanghai, 200065 China; 3 Laboratory of Biomechanics, Tongji University School of Medicine, Shanghai, 200092 China

**Keywords:** Varus ankle arthritis, Realignment surgery, Supramalleolar osteotomy, Biomechanics

## Abstract

**Background:**

Supramalleolar osteotomy (SMOT) is a well-accepted treatment method for mid-stage varus ankle osteoarthritis (OA). However, few studies have examined the role of fibular osteotomy in SMOT. The objective of the current study was to compare the biomechanical and clinical outcomes of SMOT with and without fibular osteotomy.

**Methods:**

Eight cadaveric lower legs with 10° varus/valgus SMOT models were tested using a Tekscan ankle sensor. Tibiotalar joint contact with and without fibular osteotomy conditions were compared. Forty-one varus ankle OA patients treated with SMOT were included; 22 underwent fibular osteotomy, and 19 did not. The Maryland foot score and radiological angles were used for clinical evaluation.

**Results:**

The mean contact area and pressure did not differ significantly between normal and varus/valgus conditions with the fibula preserved. After fibular osteotomy, the mean contact area decreased and the mean contact pressure increased significantly in varus and valgus conditions (*P* < 0.01). The loading center moved to the opposite direction with and without fibular osteotomy in varus/valgus conditions. After a mean follow-up of 36.6 months (range 17–61), there was no significant difference in the Maryland scores of the two groups. However, in the fibular osteotomy group, the talar tilt angle decreased (*P* < 0.05), and the tibiocrural angle improved significantly (*P* < 0.01).

**Conclusions:**

Fibular osteotomy facilitates the translation of tibiotalar contact pressure and is helpful for varus ankle realignment in patients with large talar tilts and small tibiocrural angles.

## Background

Supramalleolar osteotomy (SMOT) was first introduced by the American authors Speed and Boyd in 1936 [1] and was popularized after Takakura’s report in 1995 for the treatment of early- and mid-stage asymmetric ankle osteoarthritis (OA) [2]. Previous studies have reported that SMOT could restore the weight-bearing alignment of the ankle joint [3–5], decrease the contact pressure of the medial part of the tibiotalar joint [6], restore the congruence of the tibiotalar joint surface [5, 7, 8], improve the chondromalacia [9], postpone the OA progress [10], and even reverse the radiological ankle OA stages [2, 9, 11, 12].

However, whether a fibular osteotomy is needed in SMOT still in controversial. Some authors have proposed combined fibular osteotomy for all SMOT patients [2, 12–16], some have suggested that the fibula should always be preserved [3, 11, 17, 18], and some authors have performed fibular osteotomy depending on the conditions [4, 5, 7, 19]. Few studies have focused on the role of the fibula in SMOT. Stufkens et al. [6] reported that the tibiotalar contact force shifted in different directions with and without fibular osteotomy after SMOT. Furthermore, no clinical study has directly compared the outcomes of SMOT patients with and without fibular osteotomy. We hypothesized that (1) the tibiotalar joint contact area and stress changes differ in asymmetric ankles with and without fibular osteotomy, and (2) the patients in these groups have different clinical outcomes.

## Methods

### Biomechanical study

Eight fresh-frozen cadaveric lower legs with knee joints donated to our hospital were used for the biomechanical study. The limbs were thawed at room temperature for at least 24 h before the test. Legs with malalignment and degeneration of the ankle joints were excluded radiologically.

The knee joint was fused using three 3.5-mm Steinmann pins. All of the soft tissues were removed except the knee and ankle ligaments and the interosseous membrane. Preconditioning cyclical loading was performed 10 times with a load of 600 N using a load frame (Changchun Mechanical Science Research Institute Co., Ltd., China) to absorb the plastic deformation of the lower leg. Pressure measurements were obtained using a Tekscan 5027 ankle sensor (Tekscan, Inc., South Boston, USA). The total matrix area of the ankle sensor was 784 mm^2^ (28 mm × 28 mm) with 1936 sensels. Previous studies have shown high repeatability of Tekscan system results [20–22].

**Fig. 1:**
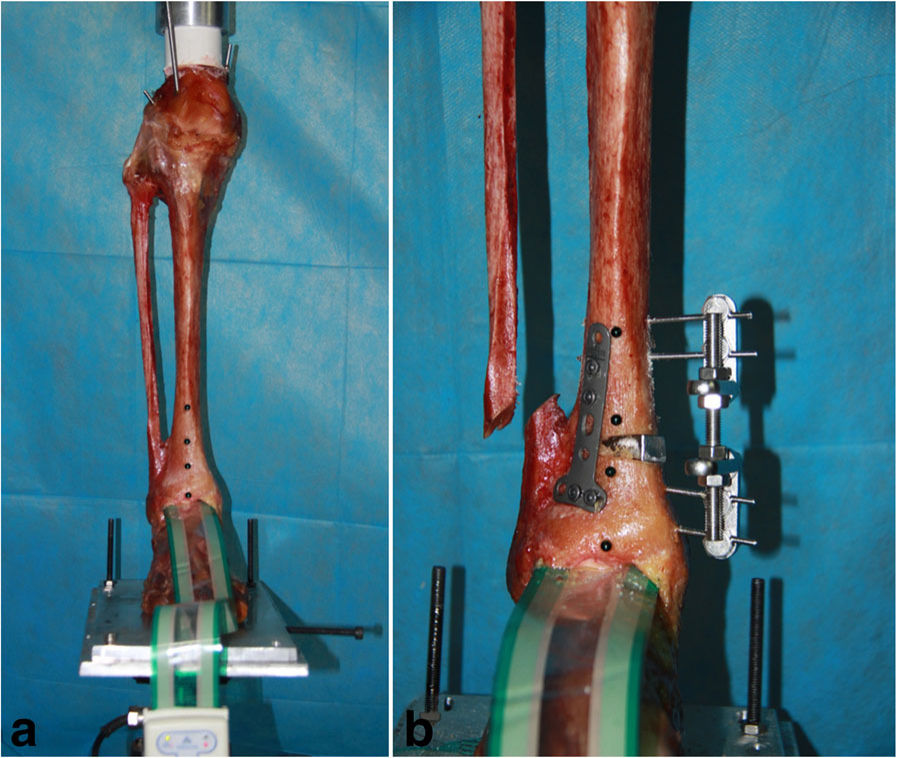
Biomechanical test of the normal tibiotalar joint contact condition using a Tekscan ankle sensor inserted into the anterior joint space (**a**). An aluminum wedge was inserted to create the distal tibial articular valgus deformity (**b**)

The sensor was gently placed anteriorly into the tibiotalar joint space. The load was increased to 600 N, maintained for 2 s, and then decreased to 50 N. Tibial osteotomy was approximately 5 cm proximal to the medial malleolar tip, and a 10° aluminum wedge was used to create the 10° valgus deformity (Fig. 1). A 10° closing wedge osteotomy was made to create the 10° varus deformity. The tibiotalar joint contact was recorded using Tekscan 32-bit software (Tekscan, Inc., South Boston, USA) for each condition with 600 N loading. The contact area and contact pressure of each loading were recorded for analysis.

### Clinical series

The clinical study was approved by the ethics committee of Honghui Hospital. The authors retrospectively analyzed the outcomes of SMOT with or without fibular osteotomy for the treatment of varus ankle arthritis between April 2009 and October 2013. The inclusion criteria were as follows: (1) adult patients with a tibial articular surface (TAS) angle less than 84°, (2) with stage 2 or stage 3 symptomatic ankle OA according to the modified Takakura ankle OA classification [12], and (3) treated with SMOT and with at least 1 year of follow-up.

A total of 41 patients (14 males and 27 females) were included. The mean age was 50.7 years (range 32–71 years). According to the modified Takakura ankle OA classification, there were 14 stage 2 patients, 19 stage 3A patients, and 8 stage 3B patients. Twenty-two patients had undergone fibular osteotomy and 19 had not. There was no significant difference in the basic information between the two groups (Table 1).

**Table 1:** Basic information of included patients (mean ± SD, *n* = 41)

	Fibular preserved (*n* = 19)	Fibular osteotomy (*n* = 22)	*P* value
Cases/M/F	19/9/10	22/5/17	0.32
Age (year)	48.8 ± 14.5	52.4 ± 8.9	0.34
Stage 2/3	7/12	5/17	0.32
Union time (months)	3.6 ± 0.4	3.9 ± 0.7	0.11
BMI	26.6 ± 0.6	26.3 ± 0.5	0.09
Smoking	4	5	0.89
Diabetes	2	2	0.88
OA/TOA	9/10	10/12	0.90

*M/F* male/female, *BMI* body mass index, *OA* osteoarthritis, *TOA* traumatic osteoarthritis

### Surgical technique

All of the included patients were treated with medial tibial opening wedge osteotomy, which has been well described in the literature [2, 12, 16, 23]. For those who did not undergo fibular osteotomy, a single medial-anterior longitudinal approach medial to the anterior tibial tendon was used. Cartilage debridement or microfracture was performed when the patient had a cartilage lesion. The tibial osteotomy was made approximately 5 cm proximal to the medial malleolar tip. A K-wire was placed from the medial to the lateral cortex to guide the osteotomy. The lateral cortex was carefully preserved. If an intraoperative lateral cortex fracture occurred, a plate or staple was used for fixation. According to the preoperative plan, the aim was a TAS angle of 90° to 92°. Iliac autograft or allograft or β-tricalcium phosphate was used to fill the tibial osteotomy site according to the patient’s preference. β-tricalcium phosphate was used in some of our initial patients but was discontinued because of its longer union time. The osteotomy site was internally fixed with a medial plate. If the tibiocrural (TC) angle was decreased more than 5° compared with the contralateral side or the fibula presented a rotational deformity or interfered with the reduction of the tibial plafond and talus, a fibular osteotomy was performed with a lateral approach. The alignment of the ankle joint and the position of the talus in the mortise were verified fluoroscopically before and after final fixation.

### Radiographic and functional evaluation

**Fig. 2:**
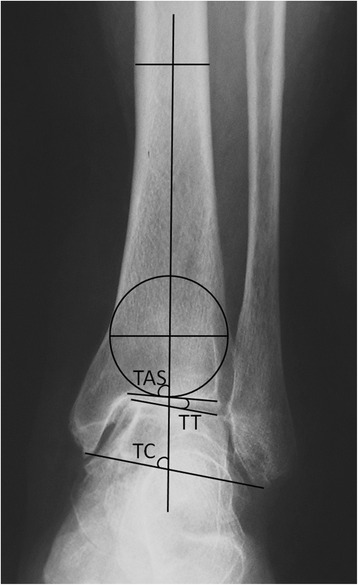
Anterior-posterior view of the ankle joint. *TC* tibiocrural angle, *TAS* tibial articular surface angle, *TT* talar tilt angle

The radiological evaluation included the TAS, the talar tilt (TT) angle, and the TC angle on a weight-bearing anterior-posterior ankle X-ray (Fig. 2). The Maryland foot score was used to evaluate the functional outcomes pre- and postoperation [24]. To analyze the changes in the radiographic grade, stages 2, 3A, 3B, and 4 of the modified Takakura classification system were assigned quantitative scores of 2, 3, 4, and 5, respectively.

### Statistical analysis

Descriptive statistics were calculated as the mean ± standard deviation. Statistical analysis of the included data was performed using Student’s *t* test or Pearson’s chi-square test, with the level of significance set at *P* < 0.05.

## Results

### Biomechanical analysis

**Fig. 3:**
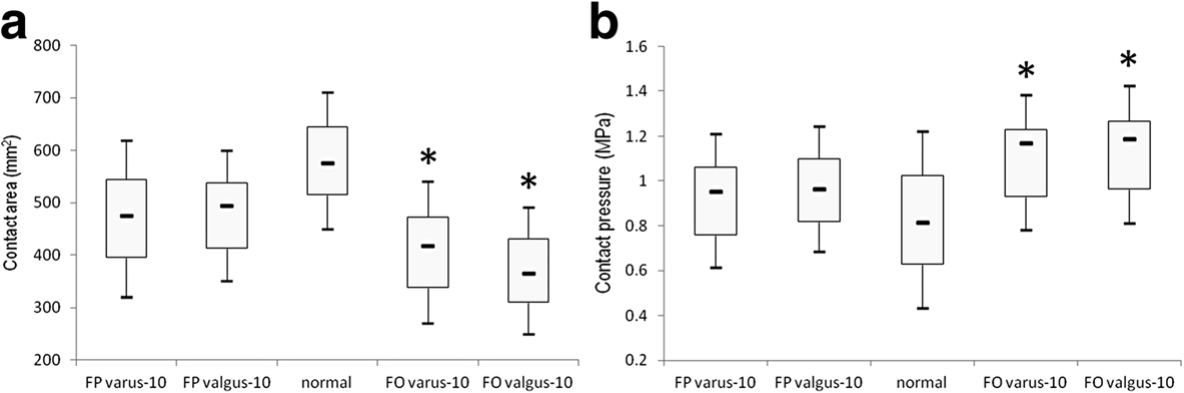
The mean contact area under different conditions (**a**). The mean contact pressure under different conditions (**b**). The *transverse line* represents the median. The *box* represents the lower to upper quartiles. The *whiskers* represent the 95 % confidence interval. *FP* fibula preserved, *FO* fibular osteotomy

The mean contact area of a normal tibiotalar joint in a neutral position with 600 N loading was 576 ± 98 mm^2^ (range 453–710), and the mean contact pressure was 0.81 ± 0.26 MPa (range 0.43–1.22). With the fibula preserved, the mean contact areas and pressures in varus and valgus conditions did not differ significantly from those of normal controls (Fig. 3). After fibular osteotomy, the mean contact area decreased, and the mean contact pressure increased significantly in varus and valgus conditions (Table 2).

**Table 2:** Biomechanical outcomes of different conditions (mean ± SD, *n* = 8)

		Fibular preserved	*P* value^a^	Fibular osteotomy	*P* value^a^
Area (mm^2^)	Varus	475 ± 99	0.06	418 ± 94	0.01
	Valgus	495 ± 90	0.11	36 4± 103	0.00
Pressure (MPa)	Varus	0.96 ± 0.21	0.23	1.17 ± 0.21	0.01
	Valgus	0.95 ± 0.20	0.25	1.19 ± 0.22	0.01

^a^Compare with the normal condition

**Fig. 4:**
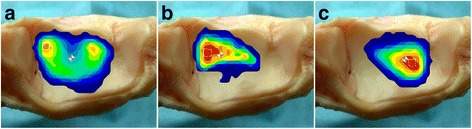
Tibiotalar joint contact pressure distribution on the normal joint (**a**), with the loading center moved laterally in a valgus deformity with fibular osteotomy (**b**), with the loading center moved medially in a varus deformity with fibular osteotomy (**c**). *Warmer colors* indicate higher pressures

Under normal conditions, two main loading zones appeared in the anterior-lateral and anterior-medial portions of the tibiotalar joint. The loading center was near the center region of the joint (Fig. 4). With the fibula preserved, the loading center moved laterally under varus conditions and moved medially under valgus conditions. However, after fibular osteotomy, the loading center moved to the opposite direction.

### Clinical outcomes

The mean follow-up time was 36.6 months (range 17–61). All of the included patients achieved bony union within a mean duration of 3.8 months (range 3–8). Union times longer than 6 months occurred in three patients for whom β-tricalcium phosphate was used to fill the osteotomy gaps. Two patients underwent ankle arthrodesis at 17 (fibular osteotomy) and 26 (fibular preserved) months because of pain and dysfunction. The Maryland foot score improved significantly (*P* < 0.01), as did the radiological angles and Takakura stage (Table 3).

**Table 3:** Pre- and postoperative clinical outcomes (mean ± SD, *n* = 39)

	Preoperation	Postoperation	*P* value
Maryland	58.3 ± 12.0	81.6 ± 6.0	0.00
TAS	81.2 ± 3.0	88.3 ± 2.5	0.00
TT	5.4 ± 4.1	2.2 ± 1.7	0.00
TC	75.6 ± 4.2	83.0 ± 2.9	0.00
Takakura stage	2.8 ± 0.7	2.3 ± 0.9	0.01

*TAS* tibial anterior surface, *TT* talar tilt angle, *TC* tibiocrural angle

No significant difference in the functional outcomes or the Takakura stage improvement rate was found between the patients with and without fibular osteotomy (Table 4). However, the TT of the fibular osteotomy group decreased from 6.6° ± 4.7° (range 0°–16°) to 2.2° ± 1.8° (range 0°–8°), and that of the preserved-fibula group decreased from 4.2° ± 2.8° (range 0°–11°) to 2.2° ± 1.4° (range 0°–5°); the decrease was significantly greater in the fibular osteotomy group (*P* = 0.02). Additionally, the TC angle was significantly improved in the fibular osteotomy group (*P* < 0.01).

**Table 4:** Improvement of clinical outcomes (mean ± SD, *n* = 39)

	Fibular preserved (*n* = 18)	Fibular osteotomy (*n* = 21)	*P* value
Maryland	24.9 ± 10.7	22.1 ± 11.5	0.44
TAS	8.7 ± 2.5	9.2 ± 2.8	0.56
TT	2.2 ± 1.5	4.4 ± 3.6	0.02
TC	6.6 ± 2.8	9.8 ± 3.5	0.01
Takakura stage^a^	50 %	62 %	0.46

*TAS* tibial anterior surface, *TT* talar tilt angle, *TC* tibiocrural angle

^a^The decrease rate of modified Takakura stage

## Discussion

Ankle OA is a degenerative disease with a high rate of angular deformity of the distal tibial articular surface. Valderrabano et al. [25] reported that 55 % of patients with ankle OA presented with varus malalignment, whereas 8 % had valgus malalignment. It is well known that uneven pressure on the articular surface of the lower extremities is closely related to the degeneration of cartilage, which may induce and accelerate the progress of OA [26]. Realignment surgery based on this theory aims to redistribute the joint’s weight-bearing line and unload the degenerated articular surface onto more viable cartilage to delay the progress of OA.

Biomechanical studies suggest that distal tibial deformities are responsible for the contact pressure changes of the tibiotalar joint [1, 27]. Tarr and colleagues reported that distal tibial articular deformities with an angulation of 15° in the sagittal plane showed a 42 % decrease in the contact area [27]. Stamatis and colleagues found that a 10° valgus supramalleolar osteotomy decreased 42 % of the force on the medial talar dome [1]. Stufkens et al. [6] reported that the mean reduction in contact area was up to 36 % in cases with a 15° valgus deformity. According to the current study, the mean contact area decreased 18 % in 10° varus deformities and 14 % in 10° valgus deformities with the fibula preserved. However, after fibular osteotomy, the mean contact area decreased up to 27 % in 10° varus deformities and 37 % in 10° valgus deformities. Additionally, the mean contact pressure increased from 19 to 44 % under varus conditions after fibular osteotomy and from 17 to 47 % under valgus conditions. This difference likely arises because the fibular and relative lateral ligaments and syndesmotic ligaments prevented the changes in tibiotalar joint contact and stress transfer. Additionally, we agree with Becker and Myerson that the ability of the ankle and foot to tolerate deformity above the ankle joint depends on the flexibility and the ability of the foot to accommodate and compensate for the deformity [28].

Since Takakura’s report in 1995 [2], the published evidence for the use of SMOT as an alternative treatment for persistent painful asymmetric ankle OA increased rapidly during the last two decades [29]. The results showed good short- to mid-term outcomes for pain relief, functional improvement, and the resumption of sports and recreation activities [2–5, 7–9, 11, 12, 14–18, 26]. According to the current results, the functional and radiological outcomes all improved significantly (Table 3). The Maryland foot score was used in the current study because it assigned more points to pain and gait, which were more correlated with the patients’ general activity [5].

During the SMOT procedure, whether and when a fibular osteotomy is needed remains unclear. In previous studies, the authors recommended fibular osteotomy as a standard procedure for the treatment of varus ankle OA [2, 12, 16]. However, some studies recommended preserving the fibula in all cases [3, 11, 17, 18]. We based the decision to perform the fibular osteotomy on the pre- and intraoperative evaluation. When the TC angle is decreased more than 5° compared with the contralateral side, which indicates a longer fibula or varus change, a fibular osteotomy may facilitate the reduction of the tibial plafond and talus [19]. Stufkens et al. [6] reported that creating a supramalleolar valgus deformity would not cause a shift in contact towards the lateral side of the tibiotalar joint, and the restricting role of the fibula was revealed when an osteotomy was performed. Our biomechanical study confirmed their results and showed that the tibiotalar joint’s loading center moved in opposite directions with and without fibular osteotomy.

**Fig. 5:**
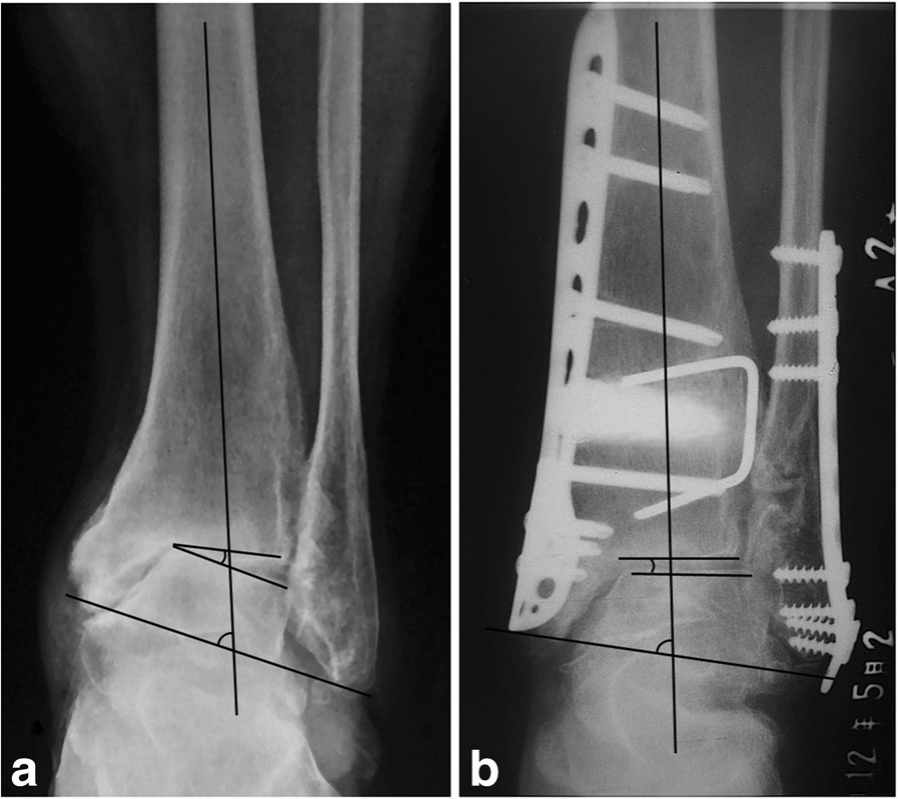
Anterior-posterior view of an ankle joint with supramalleolar osteotomy and fibular osteotomy. The talar tilt angle was completely corrected, which was decreased from 14.5° preoperation (**a**) to 1.2° postoperation (**b**)

**Fig. 6:**
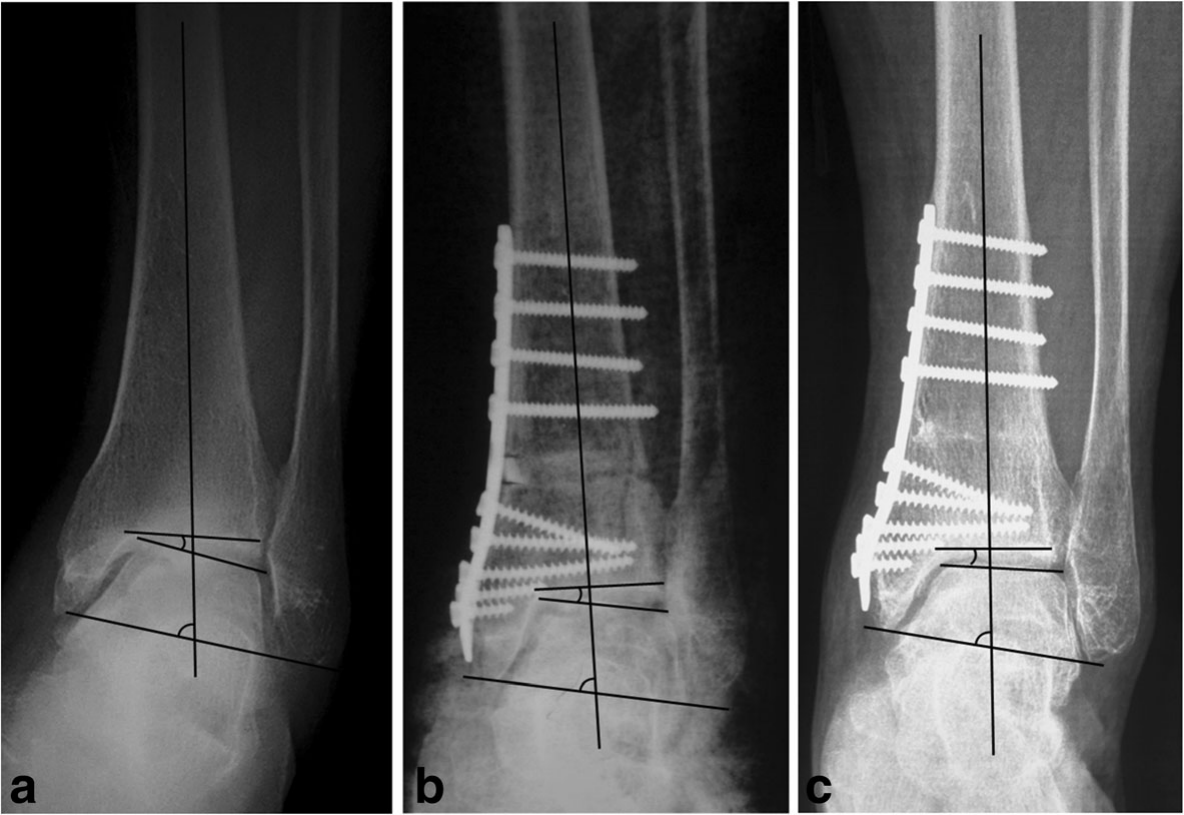
The preoperative talar tilt (TT) angle was 10.7° (**a**), and the postoperative TT angle was corrected to 7.8° (**b**); however, the TT angle had decreased to 1.5° at the 32-month follow-up (**c**)

According to a current study, in patients with fibular osteotomy (Fig. 5), the TT decreased more than in those without fibular osteotomy (*P* = 0.02). The role of TT in SMOT is highly controversial. Some authors have reported a significant decrease of the TT [4, 7, 8, 11, 17]; however, others have not [3, 12, 15, 19]. Tanaka et al. [12] reported that in all patients with a preoperative TT greater than 10°, the joint space did not return to normal. Lee et al. [15] reported that the preoperative TT was correlated with the postoperative TT and suggested that an optimal preoperative threshold for predicting high postoperative TT was 7.3°. Mann et al. [18] reported that the clinical results were good, although the mean postoperative TT was 10°. Additionally, Kim et al. [11] reported that no radiological outcome seemed to have a significant influence on the clinical outcomes. We agree with Mann et al. [18] that realignment surgery of the ankle joint with osteotomy is worthwhile, even for high TT patients, because the osteotomy will redistribute the contact forces of the ankle joint and move the mechanical axis point of the ankle laterally [13], prolong the viability of a more normally aligned joint, and even restore the TT to a neutral position (Fig. 6). We agree that the radiological changes need time to appear. Cheng et al. [9] reported that all patients presented opened medial joint spaces that gradually increased over the follow-up time. It was 1 year after SMOT before the radiographic evidence showed that the joint space had widened enough to demonstrate regeneration of the arthritic ankle, and after that point, improvement seemed to continue year by year [9]. However, in the current study, the mean TT degrees postoperatively were similar between the two groups, which might suggest that the patients in the fibular osteotomy group had a larger TT preoperatively. Additionally, we found a significant improvement of the TC angle in the fibular osteotomy group.

We agree that not all SMOT patients require fibular osteotomy. If the varus ankle OA patient presents with a widened mortise, SMOT with fibular preservation may be a good choice [3]. The authors reported that the center of the talus moved laterally, with a mean migration of 5.7 mm (*P* < 0.01) [3]. Additionally, the fibula should be preserved if the patient has normal lateral anatomic construction, and the ankle joint alignment was corrected to a satisfactory degree during the operation.

The limitations of current study include that only 10° angular deformities were evaluated because we just wanted to evaluate the difference in tibiotalar joint contact conditions between fibular preservation and osteotomy. Additionally, the use of fewer conditions could decrease the error between different loading times. The limitations of using a static cadaver model were also present. The limitation of our clinical study contained the follow-up duration, the retrospective design, and the lack of information regarding intraarticular changes. Although the outcomes will change with time, our early results confirm that the functional outcomes of SMOT were good in terms of pain relief and correction of malalignment, even in patients with high TT angles. Additionally, in appropriate cases, fibular osteotomy was helpful for translating contact pressure and restoring joint congruence. Other limitations included that we only used the Maryland foot score for functional evaluations and that the pre- and postoperative radiological evaluations did not include the weight-bearing full leg anterior-posterior view, which would be useful for the full leg alignment evaluation. Haraguchi et al. [13] used hip-to-calcaneus radiographs to evaluate lower limb alignment in SMOT patients and reported that when the preoperative mechanical axis point was more medial than the tibial plafond, the point was insufficiently moved to the lateral side, and the clinical outcomes were less satisfactory. This finding is important for preoperative evaluations and selecting the proper operation for mid-stage varus ankle OA patients.

## Conclusions

In conclusion, supramalleolar osteotomy is recommended for the treatment of early- and mid-stage varus ankle OA and results in substantial functional improvement and malalignment correction. Fibular osteotomy can facilitate the tibiotalar contact pressure translation and is helpful for varus ankle joint OA realignment in patients with large TT and small TC angles.

## Abbreviations

FO: Fibular osteotomy
FP: Fibular preserved
OA: Osteoarthritis
SMOT: Supramalleolar osteotomy
TAS: Tibial articular surface
TC: Tibiocrural
TT: Talar tilt
